# Untargeted Lipidomic Profiling Reveals Distinctive Lipid Features in Three Cultivated *Fritillaria* Species

**DOI:** 10.3390/plants15182782

**Published:** 2026-09-11

**Authors:** Guangbo Xie, Dinghui Liu, Shan Luo, Weiqiang Fan

**Affiliations:** 1 School of Life Science and Technology, University of Electronic Science and Technology of China, Chengdu 610054, China; liudinghui2022@163.com (D.L.); luoshan_2001@163.com (S.L.); 2 Aba Prefecture Institute for Food and Drug Control, Ma′erkang 624000, China; 815193112@qq.com

**Keywords:** *Fritillaria*, untargeted lipidomics, UPLC-Q-TOF-MS, metabolic phenotyping, FAHFA, triacylglycerol (TAG)

## Abstract

Fritillariae Cirrhosae Bulbus (Chuanbeimu) is a high-value traditional Chinese medicine with significant botanical diversity. While extensive research has focused on its alkaloid components, the global lipidome, which is vital for plant stress adaptation and signaling, remains largely unexplored. This study employed untargeted lipidomics via UPLC-Q-TOF-MS to characterize the lipid profiles of three dominant cultivated materials: *Fritillaria unibracteata*, *F. cirrhosa*, and *F. unibracteata* var. *wabuensis*. A conserved qualitative lipidome encompassing 311 lipid species was identified across all samples. Interestingly, *Fritillaria* bulbs exhibited a uniquely “precursor-rich” profile dominated by free fatty acids (37.23–47.12%) and phosphatidic acids (6.19–11.33%), highlighting extensive lipid turnover potentially influenced by both alpine environmental adaptation and post-harvest processing. Chemometric analyses revealed that despite qualitative similarities, the three cultivated groups possessed highly distinct quantitative lipid phenotypes. A panel of 97 differentially accumulated lipids was identified, among which specific triacylglycerols (TAGs) and fatty acid esters of hydroxy fatty acids (FAHFAs) exhibited the most pronounced stepwise variations. Pathway analysis highlighted significant variations in glycerolipid and glycerophospholipid metabolism among the specific sample groups. By acknowledging the confounding effects of specific agricultural microclimates and post-harvest factors, this exploratory study provides the first comprehensive phytochemical baseline for the *Fritillaria* lipidome, offering novel insights into their biochemical diversity and providing structural candidates for future targeted research into their adaptive lipid remodeling and pharmacological potential.

## 1. Introduction

Fritillariae Cirrhosae Bulbus (Chinese: *Chuanbeimu*), derived from the dried bulbs of *Fritillaria* species, stands as one of the most prestigious traditional Chinese medicines (TCMs), widely prescribed for its potent antitussive and expectorant properties [1,2]. Notably, recent pharmacological studies have also highlighted its profound anti-inflammatory activities, which are crucial for treating respiratory diseases [3,4]. The *Chinese Pharmacopoeia* recognizes six *Fritillaria* species, including *F. cirrhosa* D. Don, *F. unibracteata* Hsiao et K. C. Hsia, *F. przewalskii* Maxim., *F. delavayi* Franch., *F. taipaiensis* P. Y. Li, and *F. unibracteata* var. *wabuensis* (S. Y. Tang et S. C. Yue) Z. D. Liu, S. Wang et S. C. Chen, as authorized botanical sources [5]. However, driven by the severe depletion of wild resources and soaring market demand, the industry has transitioned from wild harvesting to artificial cultivation [1]. Among the authorized sources, *F. cirrhosa*, *F. unibracteata*, and *F. unibracteata* var. *wabuensis* have emerged as the dominant cultivated taxa in the Western Sichuan Plateau, the widely recognized geo-authentic (*Daodi*) region for *Chuanbeimu* [1,6,7].

To date, phytochemical investigations have unveiled a diverse chemical profile in *Fritillaria* bulbs, encompassing alkaloids, sterols, terpenoids, nucleosides, and polysaccharides [2,8,9,10,11]. Historically, research has predominantly focused on steroidal alkaloids as the primary therapeutic agents [12]. While alkaloids are undeniably critical, this alkaloid-centric perspective overlooks the complexity of the global metabolome. Specifically, lipids, which are fundamental for cellular membrane integrity and energy storage, remain largely underexplored in these species. Investigating the lipidome provides complementary insights that alkaloid profiling cannot offer. While alkaloids represent specialized downstream secondary metabolites, lipids constitute the foundational metabolic network that not only directly governs plant stress adaptation but also potentially contributes to the overarching pharmacological efficacy, such as the anti-inflammatory effects mediated by specific bioactive lipid molecules [13,14,15].

Although *Fritillaria* species are not categorized as oil-rich crops, their bulbs contain bioactive lipid components, including phytosterols and fatty acids, which are frequently detected in volatile oil fractions or general metabolic profiling [16]. Furthermore, recent systematic lipidomic investigations into related *Liliaceae* plants, specifically *Lilium* bulbs, have revealed a complex diversity of lipid classes, such as glycerophospholipids and triacylglycerols, demonstrating that bulb lipids can serve as crucial chemical markers for geographical discrimination and quality characterization [17]. Crucially, distinct lipid classes play multifaceted roles in plant adaptation to abiotic stresses, a vital trait for alpine species like *Fritillaria* cultivated in the harsh microclimates of the Qinghai–Tibet Plateau [18,19]. For instance, glycerolipids and glycerophospholipids undergo dynamic remodeling to maintain membrane fluidity and structural integrity under cold stress [20,21]. Concurrently, specific sphingolipids and sterol lipids orchestrate microdomain architecture and lipid-raft-mediated signal transduction, while free fatty acids serve not merely as metabolic intermediates but as essential precursors for rapid-response signaling molecules [22,23].

In recent years, the rapid advancement of untargeted lipidomics utilizing UPLC-Q-TOF-MS has revolutionized our ability to systematically characterize lipid diversity and dynamic membrane remodeling in plants [24]. This robust analytical strategy has been increasingly applied to medicinal crops not only to dissect complex environmental adaptation mechanisms but also to establish foundational biochemical baselines for phytochemical phenotyping and quality evaluation [25]. Despite these technological leaps, a comprehensive global lipidomic map for the *Fritillaria* genus is completely lacking. The specific quantitative variations in lipid classes among different cultivated *Fritillaria* taxa, and how their lipid architectures reflect their respective biochemical identities, represent a significant knowledge gap that hinders a holistic understanding of their metabolic diversity.

Building upon our previous successful applications of untargeted lipidomics in characterizing other characteristic regional resources, specifically *Morchella* species (Morels) [26,27] and *Zanthoxylum* species (Sichuan pepper) [28], we extended this robust analytical strategy to the *Fritillaria* genus. In this study, we systematically characterized and compared the lipid profiles of the three dominant cultivated *Fritillaria* materials (*F. cirrhosa*, *F. unibracteata*, and *F. unibracteata* var. *wabuensis*). The specific objectives were to (1) elucidate the global lipid composition and unique biochemical characteristics of these botanical sources for the first time; and (2) explore the quantitative lipidomic divergences among the three taxa to enrich our fundamental phytochemical knowledge of this highly valuable genus.

## 2. Results and Discussion

### 2.1. Global Lipidomic Characterization

#### 2.1.1. Overview of Lipid Identification and Classification

A total of 311 distinct lipid species were identified and characterized in the bulbs of *F. cirrhosa*, *F. unibracteata*, and *F. unibracteata* var. *wabuensis* (Table S1, Figures S1 and S2). Taxonomically, these lipids were classified into five major categories based on the comprehensive classification system described by Fahy et al. [29]: fatty acyls (FA), glycerolipids (GL), glycerophospholipids (GP), sphingolipids (SP), and sterol lipids (ST).

**Figure 1:**
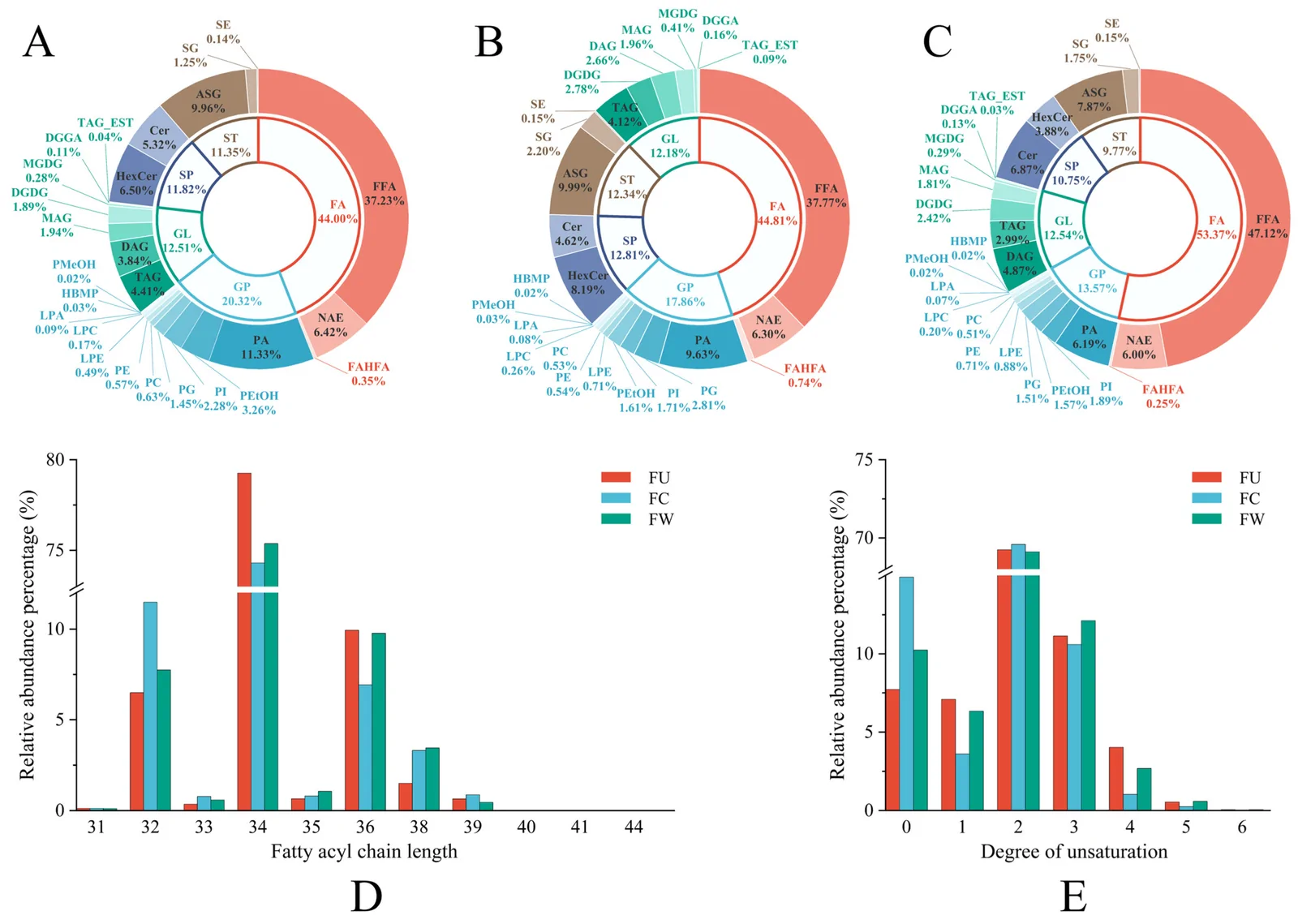
Global lipid profiles and structural characteristics in three *Fritillaria* taxa. (**A**–**C**) Double-ring charts illustrating the relative abundance of lipid categories (inner ring) and subclasses (outer ring) in *F. unibracteata* (**A**), *F. cirrhosa* (**B**), and *F. unibracteata* var. *wabuensis* (**C**). (**D**,**E**) Distribution profiles of total acyl chain lengths (carbon numbers) (**D**) and degrees of unsaturation (number of double bonds) (**E**) within diacyl glycerophospholipids across the three *Fritillaria* species. The y-axis represents the relative percentage abundance of the corresponding lipid species. Percentages in (**D**) and (**E**) were calculated based on the mean abundance values from three biological replicates. FU *F. unibracteata*, FC *F. cirrhosa*, FW *F. unibracteata* var. *wabuensis*.

In terms of molecular diversity, the GL category exhibited the highest number of identified species (91), followed by GP (67), SP (66), FA (46), and ST (41). These categories were further annotated into 26 distinct subclasses. From a quantitative perspective, however, the lipidomic landscape was predominantly composed of FAs. The total abundance of FAs ranged from 44.00% to 53.37% of the total lipid content across the three sampled groups, serving as the fundamental lipid pool. GPs constituted the second largest category (13.57–20.32%), highlighting the importance of membrane lipids in bulb physiology. The remaining categories, GLs (12.18–12.54%), SPs (10.75–12.81%), and STs (9.77–12.34%), showed comparable and moderate abundance levels (Table 1 and Figure 1A–C).

At the subclass level, the lipid profile was characterized by the dominance of free fatty acids (FFA), which accounted for 37.23% to 47.12% of the total lipids. Furthermore, substantial accumulations of phosphatidic acids (PA, 6.19–11.33%) and acylated steryl glucoside (ASG, 7.87–9.99%) were observed as the second and third most prevalent subclasses, respectively (Table 1 and Figure 1A–C).

Notably, qualitative analysis revealed that the vast majority of the 311 lipid species were consistently shared across the three sampled groups. While minor signals intermittently falling below the instrumental detection limit were imputed during data processing, no strictly group-specific lipid molecules were observed. This indicates that the *Fritillaria* materials share a highly conserved qualitative lipidome (metabolic background), whereas their distinct phenotypic traits are driven by quantitative variations.

#### 2.1.2. Compositional Profiles and Comparative Analysis

The distinct lipidomic signature of *Fritillaria* bulbs, specifically the unexpected dominance of metabolic precursors (FFA and PA) over structural lipids, presents a stark contrast to other phylogenetically related species. To contextualize the metabolic uniqueness of this profile, a descriptive comparative analysis was performed against *Lilium* bulbs (Liliaceae). However, it is important to note that cross-study comparisons are inherently limited by differences in extraction protocols and analytical platforms. Thus, this comparison primarily serves to highlight macroscopic profile differences rather than absolute quantitative divergence. Compared to its close phylogenetic relative *Lilium* (lily bulbs), *Fritillaria* displayed a remarkably divergent lipid strategy. Previous studies have established that *Lilium* bulbs are characterized by a classical membrane-stabilizing profile, with phosphatidylcholine (PC, 22.12–44.88%) and phosphatidylethanolamine (PE, 24.77–33.94%) serving as the primary subclasses [17]. In contrast, the *Fritillaria* bulbs in our study contained significantly lower proportions of PC and PE, accumulating much higher levels of free fatty acids (FFA), phosphatidic acids (PA), and acylated steryl glycosides (ASG).

This “Precursor-Rich” and “Sterol-Enhanced” profile observed in *Fritillaria* may reflect a rapid flux of lipid biosynthesis and membrane remodeling, potentially related to environmental adaptation in high-altitude habitats, as PA serves as a rapid-response signal under cold stress [22]. Furthermore, it is crucial to acknowledge that lipidomes are highly dynamic. The substantial accumulation of FFAs and PAs could also be significantly influenced by phospholipid degradation and phospholipase activities occurring during post-harvest processing (e.g., drying) and sample extraction. Since the current study focused on the final state of commercial medicinal materials without assessing the contribution of technological factors, such as comparing fresh versus stored samples under different processing protocols, this remains a methodological limitation. Future studies integrating targeted in vivo monitoring of fresh tissues are warranted to explicitly disentangle endogenous adaptive accumulation from post-harvest lipid turnover.

#### 2.1.3. Structural Characteristics of Membrane Lipids

To gain deeper insights into the physical properties of the cellular membranes, we analyzed the structural characteristics of di-acyl glycerophospholipids, including phosphatidylcholine (PC), phosphatidylethanolamine (PE), phosphatidic acid (PA), phosphatidylinositol (PI), phosphatidylglycerol (PG), phosphatidylethanol (PEtOH), and phosphatidylmethanol (PMeOH), which constitute the structural matrix of the lipid bilayer. Lyso-lipids (LPC, LPE, LPA) and complex lipids (hemibismonoacylglycerophosphate (HBMP)) were excluded from this calculation to strictly evaluate the bilayer-forming molecules.

In terms of fatty acyl chain length, the membrane lipids exhibited a highly conserved distribution pattern across the three sampled groups. The total carbon number of the acyl chains was predominantly concentrated at C34, which accounted for 74.30–79.26% of the total phospholipid pool. The second most abundant groups were C36 (6.92–9.94%) and C32 (6.49–11.47%) (Table S2 and Figure 1D). This specific enrichment of C34 species indicates that the membrane backbone of *Fritillaria* is primarily constructed from the pairing of palmitic acid (C16:0) with C18 fatty acids (such as 16:0/18:1, 16:0/18:2 or 16:0/18:3), forming a stable bilayer architecture.

Regarding the degree of unsaturation, the analysis revealed a striking dominance of polyunsaturated species. Based on the total number of double bonds per molecule, lipid species were categorized into saturated (SFA, 0 double bonds), monounsaturated (MUFA, 1 double bond), and polyunsaturated (PUFA, ≥2 double bonds). Our data showed that PUFA species constituted the overwhelming majority, accounting for 81.51–85.19% of the total membrane lipids. Specifically, species containing 2 double bonds were the most prevalent (67.98–69.59%), followed by those with 3 double bonds (10.60–12.12%), whereas fully saturated lipids (SFA) accounted for only 7.72–14.89% (Table S3 and Figure 1E).

Collectively, this specific lipid architecture provides a putative biochemical basis for adaptation to the high-altitude environment. In the freezing conditions of the Qinghai–Tibet Plateau (>3000 m), cell membranes are prone to phase transition from a fluid liquid-crystalline phase to a rigid gel phase, which can compromise membrane integrity [30]. The accumulation of high levels of PUFA introduces *cis*-double bonds into the acyl chains, creating spatial “kinks” that prevent the tight packing of lipid molecules [31], thereby potentially preserving membrane fluidity under cold stress [20]. However, as this exploratory study focuses exclusively on phytochemical profiling, future experiments incorporating direct morphophysiological and membrane tolerance measurements are required to empirically confirm the functional relationship between this unsaturation pattern and actual cold tolerance in *Fritillaria* species.

### 2.2. Multivariate Statistical Analysis of Lipidomic Profiles

To comprehensively evaluate the lipidomic variations among the three *Fritillaria* varieties, multivariate statistical analyses were performed using both unsupervised and supervised models.

**Figure 2:**
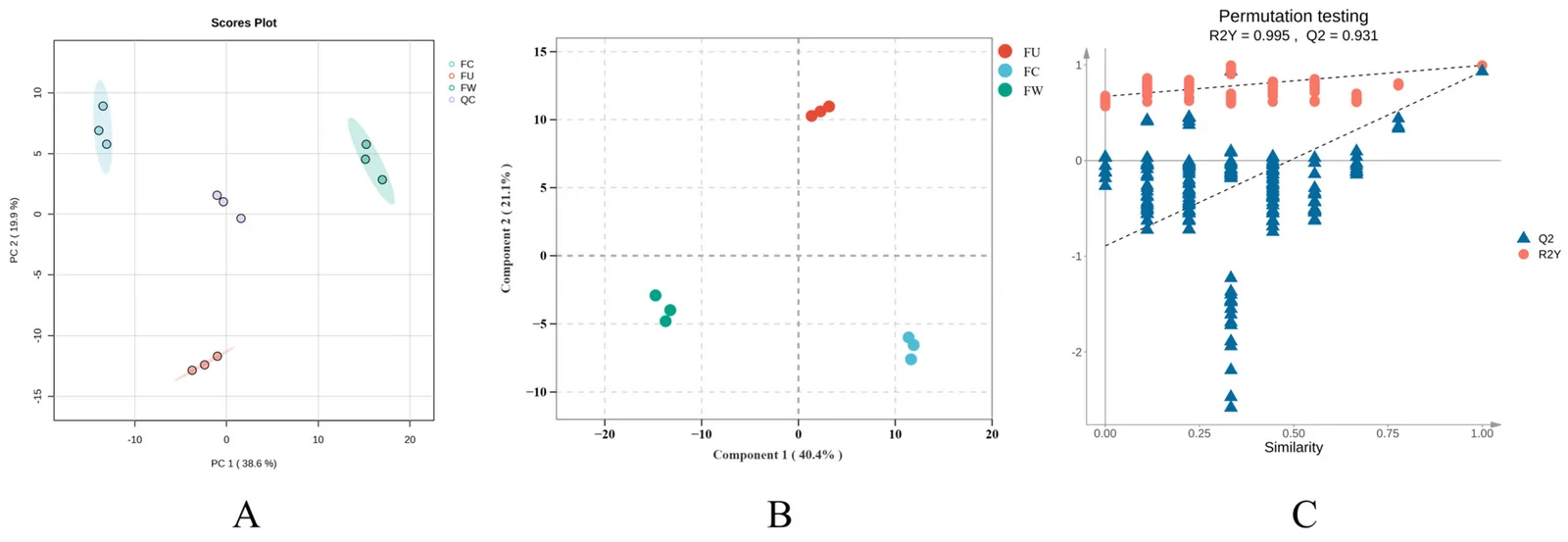
Multivariate statistical analysis of global lipidomic profiles in three *Fritillaria* taxa. (**A**) Principal component analysis (PCA) score plot encompassing the three species and quality control (QC) samples. (**B**) Partial least squares discriminant analysis (PLS-DA) score plot illustrating the complete spatial segregation among *F. unibracteata* (FU), *F. cirrhosa* (FC), and *F. unibracteata* var. *wabuensis* (FW). (**C**) Statistical validation of the established PLS-DA model via a permutation test (n = 200), confirming the model’s robustness and absence of overfitting.

First, an unsupervised Principal Component Analysis (PCA) was conducted to visualize the intrinsic sample grouping (Figure 2A). The Quality Control (QC) samples were tightly clustered in the center of the score plot, confirming the high stability and reproducibility of the analytical system. The PCA model, with the first two principal components explaining 58.5% of the total variance (PC1: 38.6%, PC2: 19.9%), revealed a clear and spontaneous separation among the three groups. A distinct triangular distribution was observed: *F. unibracteata* var. *wabuensis* (FW) was separated from the other two species along the negative PC1 axis, while *F. cirrhosa* (FC) and *F. unibracteata* (FU) were distinguished primarily along the PC2 axis. This grouping pattern demonstrates that the three sampled groups possess unique lipid phenotypes that allow for effective discrimination even without prior group information.

To further validate these differences, a supervised Partial Least Squares Discriminant Analysis (PLS-DA) was applied to the dataset (Figure 2B). The established model exhibited exceptional fitness and predictability (*R*^2^*Y* = 0.995, *Q*^2^ = 0.931), demonstrating that the model could explain 99.5% of the class variance with remarkably high predictive accuracy. Furthermore, a permutation test (n = 200) was conducted to rigorously validate the model’s robustness. As illustrated in Figure 2C, the intercepts of both *R*^2^ and *Q*^2^ were significantly lower than their original values (with the *R*^2^ intercept ideally located near or below zero), providing compelling evidence for the absence of overfitting. However, considering the relatively small sample size (n = 3 per group) compared to the high number of lipid variables, these supervised models are treated cautiously as exploratory visualizations of group separation rather than definitive predictive models. The complete and distinct segregation of the three sample groups in the PLS-DA score plot demonstrated that despite sharing a conserved qualitative lipid background, these specific cultivated materials exhibit highly distinctive quantitative lipid phenotypes.

Complementing the multivariate plots, Hierarchical Cluster Analysis (HCA) was performed to visualize the relative abundance patterns of individual lipids (Figure S3). The resulting heatmap verified high consistency among biological replicates. Consistent with the PCA results, the dendrogram revealed three independent major branches corresponding to the three botanical origins. Notably, *F. cirrhosa* (FC) and *F. unibracteata* (FU) exhibited a closer clustering relationship compared to *F. unibracteata* var. *wabuensis* (FW), implying a higher degree of lipidomic similarity between FC and FU. Distinct “block-wise” expression patterns in the heatmap highlighted specific clusters of lipids differentially accumulated in each variety.

Given that these samples were harvested from different geographic locations and altitudes typical for their respective cultivation, and varied in harvest age, these distinct clustering patterns likely reflect a combined effect of their inherent genetic background and their physiological adaptations to specific local microclimates. Therefore, the observed lipidomic divergences represent the phenotypic signatures of these specific “sampled materials” rather than strict, genetically fixed species-specific markers.

### 2.3. Identification of Distinctive Lipid Features

To precisely identify the differentially accumulated lipids responsible for the phenotypic divergence among the sampled groups, a pairwise comparison strategy was employed. Orthogonal Partial Least Squares Discriminant Analysis (OPLS-DA) models were initially constructed for each species pair (FU vs. FC, FU vs. FW, and FC vs. FW) to maximize class separation and calculate Variable Importance in Projection (VIP) scores. Differential lipids were subsequently filtered based on a rigorous combination of multivariate and univariate statistical criteria: VIP > 1.0, FDR-adjusted *p* < 0.05, and Fold Change (FC) > 2.0 or < 0.5.

**Figure 3:**
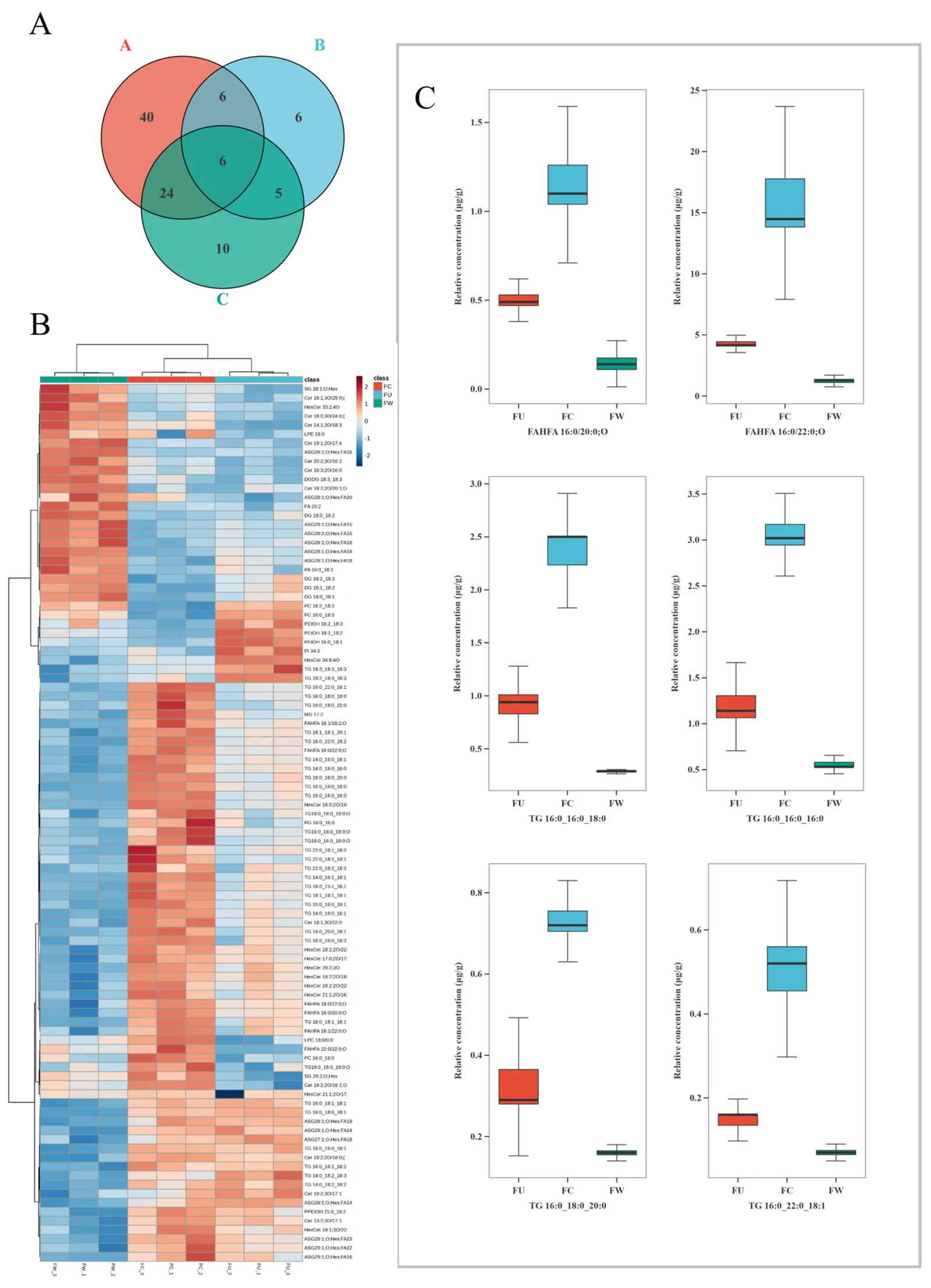
Discovery of differential lipids among the three *Fritillaria* taxa. (**A**) Venn diagram visualizing the overlap and distribution of significantly differential lipids (SDLs) derived from the three pairwise comparisons. A: FC vs. FW, B: FU vs. FC, C: FU vs. FW. (**B**) Hierarchical clustering analysis (HCA) heatmap of the 97 distinct SDLs (the union set from the Venn diagram). (**C**) Boxplots displaying the relative abundance variations in the six SDLs (the intersection set from the Venn diagram) across *F. unibracteata* (FU), *F. cirrhosa* (FC), and *F. unibracteata* var. *wabuensis* (FW). In the boxplots, the center line represents the median, box limits indicate upper and lower quartiles, and whiskers extend to 1.5 times the interquartile range.

A Venn diagram was generated to visualize the distribution and overlap of these screened differential lipids across the different pairwise comparisons (Figure 3A). Notably, the quantity of differential lipids in each comparison perfectly mirrored the macroscopic spatial distribution observed in the PLS-DA score plot (Section 2.2). Specifically, the closer spatial proximity between *F. unibracteata* (FU) and *F. cirrhosa* (FC) corresponded to the fewest differential lipids (23 lipids, Table S4), reflecting their high overall lipidomic similarity. Conversely, comparisons involving the distinctly separated *F. unibracteata* var. *wabuensis* (FW) yielded much higher numbers of differential markers, with 76 lipids for FC vs. FW (Table S5) and 45 lipids for FU vs. FW (Table S6). This quantitative alignment strongly corroborates their underlying phenotypic divergence and the robustness of the multivariate models.

By combining significantly differential lipids from all pairwise comparisons (taking the union), a comprehensive pool of 97 distinct lipid species was established (Table S7). To visualize the global variation patterns of these discriminative features, a hierarchical clustering analysis (HCA) heatmap was constructed (Figure 3B). The heatmap clearly demonstrated that the abundance profiles of these 97 lipids successfully and completely segregated the samples into three major clades corresponding to their specific botanical origins. This robust differential lipid pool not only confirms the efficacy of our screening strategy but also lays a crucial foundation for exploring their underlying biological mechanisms and metabolic pathways (as detailed in Section 2.4).

To further refine this pool and identify the most pronounced variable features, an intersection analysis highlighted six lipids (two FAHFAs and four specific TAGs) that exhibited universal and significant abundance variations across all comparisons (Figure 3C). In plant tissues, the significant accumulation of these specific TAGs likely acts not only as transient energy reservoirs but also as dynamic buffers to sequester toxic lipid intermediates, thereby regulating cellular energy homeostasis under environmental stress [32,33]. Meanwhile, trace-level lipid species like FAHFAs are increasingly recognized for their participation in fine-tuned lipid remodeling and specialized metabolic regulation [15,34]. The distinct accumulation patterns of these lipids highlight them as promising biochemical candidates for future investigations into the environmental adaptability and physiological regulation of the *Fritillaria* genus.

Importantly, the discovery of these specific lipid signatures also offers a novel perspective on the comprehensive pharmacological value of *Chuanbeimu*. While steroidal alkaloids are the historically established antitussive agents, the therapeutic potential of the lipid fraction has been largely overlooked. Notably, minor specific lipids identified herein, particularly fatty acid esters of hydroxy fatty acids (FAHFAs) and acylated steryl glycosides (ASGs), have recently emerged as potent bioactive mediators with profound anti-inflammatory properties [34,35,36]. Although present at trace levels, lipid mediators such as FAHFAs are known to exert robust physiological and anti-inflammatory responses even at minimal concentrations [37]. Since respiratory tract inflammation is a core pathological driver of chronic cough, we hypothesize that the unique accumulation of these highly active, anti-inflammatory lipid species might act synergistically with alkaloids, contributing to the overall therapeutic efficacy of *Fritillaria* bulbs. Future targeted lipidomic and pharmacological studies are warranted to empirically validate this hypothesis.

### 2.4. Metabolic Pathway Analysis and Biological Implications

To holistically explore the biological pathways potentially associated with the observed lipidomic variations, the 97 differentially accumulated lipids were subjected to KEGG pathway enrichment analysis via MetaboAnalyst 6.0. Given the structural complexity of intact lipid species, they were mapped to their corresponding generic KEGG identifiers to perform the topological analysis against the default botanical reference metabolome (Table S8).

**Figure 4:**
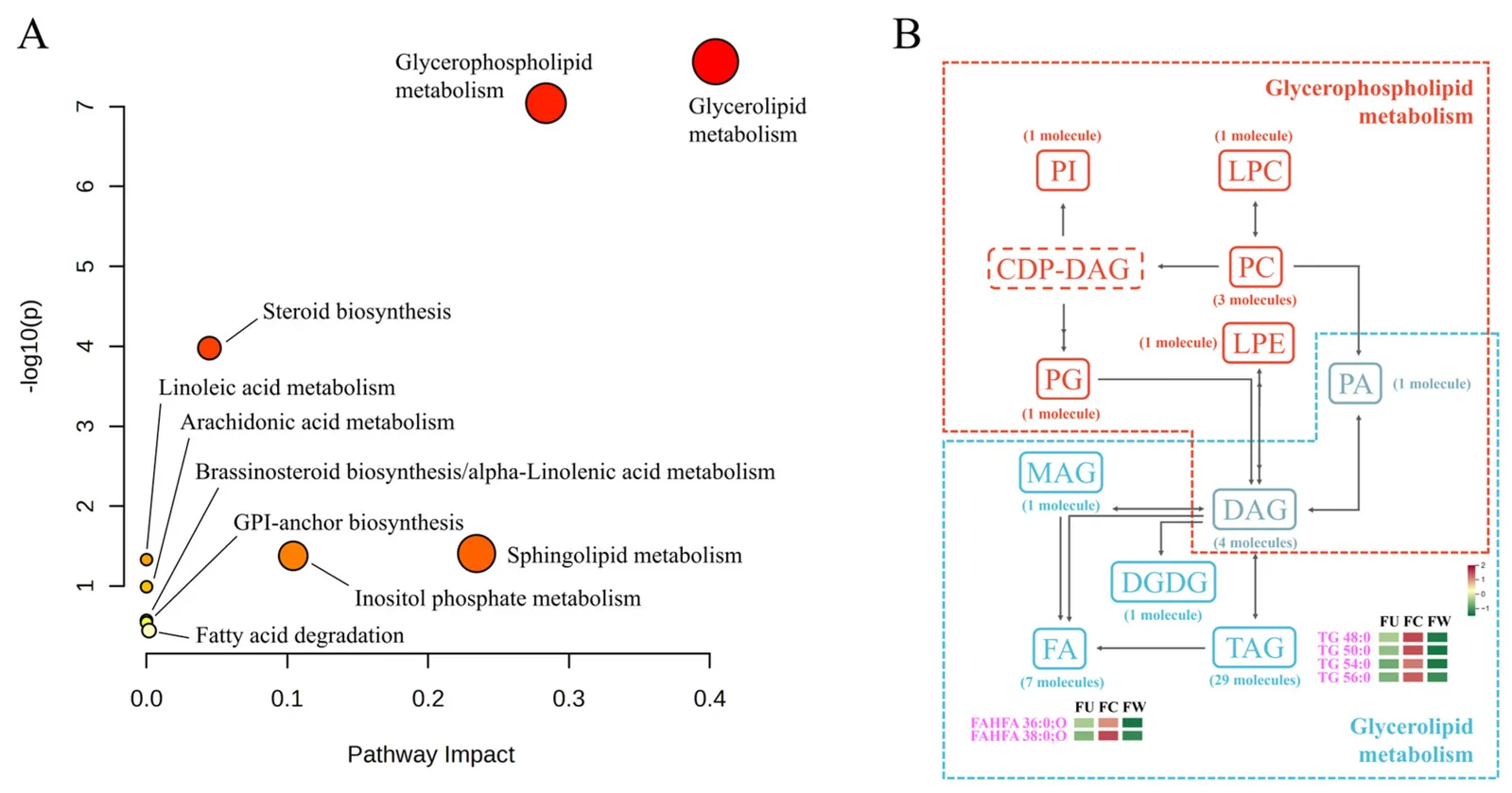
KEGG metabolic pathway enrichment and integrated topological network analysis. (**A**) Bubble chart illustrating the pathway impact and statistical significance of the 97 differential lipids among the three *Fritillaria* taxa. Bubble size is proportional to the number of differential lipids mapped to each specific pathway. The color gradient reflects the statistical significance (FDR-adjusted *p*-value), and the x-axis represents the topological impact score. (**B**) Comprehensive metabolic network map integrating the two most significantly perturbed core pathways: glycerolipid metabolism and glycerophospholipid metabolism. Numbers in parentheses located above or below the lipid metabolites at the metabolic nodes indicate the number of differential lipid hits mapped to that specific metabolite class during the enrichment analysis. Color-coded heatmaps adjacent to the six core differential lipids display their relative abundance variations across the three species, with dark red indicating the highest relative abundance and dark green representing the lowest. Metabolite classes enclosed in dashed boxes represent crucial pathway nodes that are not included among the 97 significantly differential lipids.

To rigorously control for false positives associated with multiple hypothesis testing, the False Discovery Rate (FDR) was evaluated alongside raw *p*-values. The resulting pathway impact analysis (Figure 4A) revealed that the distinct lipidomic signatures of the three sampled groups are primarily associated with the significant perturbation of two core metabolic networks (FDR < 0.01). Although sphingolipid metabolism exhibited a high topological impact (0.234), it failed to reach statistical significance after multiple testing correction (FDR = 0.716). Instead, the most highly enriched pathways exhibiting both exceptional statistical significance and topological relevance were glycerolipid metabolism (FDR = 2.53 × 10^−6^, Impact = 0.404) and glycerophospholipid metabolism (FDR = 4.18 × 10^−6^, Impact = 0.284).

As illustrated in the comprehensive metabolic network (Figure 4B), the pronounced enrichment in glycerolipid metabolism perfectly corroborates the highly variable abundances of specific triacylglycerols (TAGs). Rather than reflecting differences in bulk energy storage, this pathway enrichment further highlights the extensive lipid remodeling strategies occurring in *Fritillaria* bulbs. Concurrently, the mapped lipid intermediates in glycerophospholipid metabolism (Figure 4B) reflect distinct membrane lipid homeostasis mechanisms. It must be emphasized, however, that this KEGG pathway enrichment provides exploratory, indirect biochemical insights. The highlighted networks represent putative metabolic trajectories underlying the quantitative phenotypic divergence of these specific materials, which require future validation via transcriptomics or stable isotope tracing.

## 3. Materials and Methods

### 3.1. Chemicals and Reagents

Dichloromethane (CH_2_Cl_2_) and methanol (MeOH) of analytical grade were purchased from Merck (Rahway, NJ, USA). Acetonitrile (ACN), isopropanol (IPA), and ammonium acetate of LC-MS grade were obtained from Sigma-Aldrich (St. Louis, MO, USA). Ultrapure water was produced by a Milli-Q system (Bedford, MA, USA). Lipid standards, including phosphatidylcholine (PC 15:0_18:1(d7)), lysophosphatidylcholine (LPC 18:1(d7)), phosphatidylethanolamine (PE 15:0_18:1(d7)), lysophosphatidylethanolamine (LPE 18:1(d7)), phosphatidylglycerol (PG 15:0_18:1(d7)), phosphatidylinositol (PI 15:0_18:1(d7)), phosphatidylserine (PS 15:0_18:1(d7)), triacylglycerol (TG 15:0_18:1(d7)_15:0), diacylglycerol (DG 15:0_18:1(d7)), monoacylglycerol (MG 18:1(d7)), Cholesteryl Ester 18:1(d7), sphingomyelin (SM d18:1_18:1(d9)), and C15 ceramide-d7 were purchased from Avanti Polar Lipids (Alabaster, AL, USA). Palmitic acid (C16:0 d4) were obtained from Sigma-Aldrich (St. Louis, MO, USA). A mixed methanol solution containing all lipid standards at 5 μg/mL each was prepared and stored at –20 °C.

### 3.2. Plant Materials

Fresh bulbs of three *Fritillaria* species were harvested from their respective geo-authentic cultivation bases in Aba Tibetan and Qiang Autonomous Prefecture, Sichuan Province, China, on October 22, 2024. The samples included: (1) Four-year-old bulbs of *F. unibracteata* var. *wabuensis* collected from Fubu Village, Shaba Town, Mao County (31°50′39″ N, 103°35′13″ E; altitude 3000 m; Voucher No. FW-202401); (2) Four-year-old bulbs of *F. unibracteata* collected from Hanpan Village, Chuanzhusi Town, Songpan County (32°53′44″ N, 103°37′44″ E; altitude 3289 m; Voucher No. FU-202401); (3) Five-year-old bulbs of *F. cirrhosa* collected from Huaniu Village, Meiwo Town, Xiaojin County (30°58′1″ N, 102°11′44″ E; altitude 3400 m; Voucher No. FC-202401).

All samples were randomly collected within each cultivation base to ensure representativeness. For each species, three independent biological replicates were prepared. To account for intra-population biological variance, each biological replicate was formulated by pooling bulbs collected from at least five distinct individual plants within the same plot. The botanical origin of all samples was authenticated by Chief Pharmacist Weiqiang Fan from the Aba Prefecture Institute for Food and Drug Control. The corresponding voucher specimens were deposited at the herbarium of the institute. It should be noted that these specific sampling sites, altitudes, and harvest ages were selected to represent the typical commercial maturity and widely accepted cultivation practices for these specific taxa in their respective authentic microclimates.

Immediately after harvesting, the fresh bulbs were cleaned with distilled water to remove soil and debris. Subsequently, the samples were lyophilized (BLK-FD-5, Bolaike, Changzhou, China) to constant weight and stored at –80 °C until lipid extraction (Figure S4).

### 3.3. Lipid Extraction and Sample Preparation

A comprehensive schematic overview of the entire experimental and analytical workflow, from sample preparation and lipid extraction to instrumental analysis and bioinformatics processing, is illustrated in Figure S5. Lipid extraction was performed using a modified Folch method [38,39]. Briefly, the lyophilized bulbs were ground into a fine powder under liquid nitrogen. Approximately 15 mg of the powder was weighed into a glass centrifuge tube. Subsequently, 160 μL of internal standard solution (5 μg/mL) was added, followed by the addition of 2 mL of dichloromethane (CH_2_Cl_2_) and 2 mL of methanol (MeOH). The mixture was vortexed for 1 h at room temperature. Then, an additional 2 mL of CH_2_Cl_2_ and 1.6 mL of ultrapure water were introduced. After further vortexing and centrifugation at 5000 rpm for 10 min, the lower organic phase containing total lipids was collected and dried under a gentle stream of nitrogen. The residue was redissolved in 800 μL of CH_2_Cl_2_/MeOH (1:1, *v*/*v*) and filtered through a 0.22 μm organic membrane filter prior to LC-MS analysis.

### 3.4. UPLC-Q-TOF-MS Instrumentation and Conditions

Chromatographic separation was carried out on an UPLC-30A system (Shimadzu, Kyoto, Japan) coupled with a Kinetex C18 column (100 × 2.1 mm, 2.6 μm; Phenomenex, Torrance, CA, USA). The column temperature was maintained at 60 °C, and the autosampler was kept at 4 °C. A binary solvent system was employed for elution, consisting of mobile phase A (H_2_O/MeOH/ACN, 1:1:1, *v*/*v*/*v*) and mobile phase B (IPA/ACN, 5:1, *v*/*v*), both supplemented with 5 mM ammonium acetate. The flow rate was set at 0.4 mL/min with an injection volume of 3 μL. The gradient elution program was optimized as follows: 0–0.5 min, 80% A; 0.5–1.5 min, 80–60% A; 1.5–2 min, 60–40% A; 2–10 min, 40–5% A; 10–12 min, isocratic at 5% A; 12–13.1 min, 5–80% A; and 13.1–14 min, equilibration at 80% A.

Mass spectrometric detection was performed using a TripleTOF 6600 system (AB Sciex, Concord, ON, Canada) equipped with an electrospray ionization (ESI) source operating in both positive and negative ion modes. Data acquisition was conducted using the information-dependent acquisition (IDA) mode for simultaneous MS and MS/MS collection. The source parameters were set as follows: ion spray voltage, +5500 V (positive) and −4500 V (negative); source temperature, 600 °C; curtain gas, 35 psi; and ion source gases 1 and 2, both at 50 psi. The mass range was scanned from *m*/*z* 100 to 1200.

To guarantee the stability and reproducibility of the analytical system, a pooled quality control (QC) sample was generated by combining equal aliquots of all individual extracts. Prior to the formal data acquisition sequence, the analytical system was equilibrated with five consecutive injections of the QC sample. Throughout the analytical sequence, this QC sample was interspersed every five experimental samples, immediately followed by a blank solvent injection to monitor potential sample carryover. Crucially, all experimental samples were injected in a completely randomized order to mitigate any potential instrumental drift and batch effects.

### 3.5. Data Processing and Lipid Identification

Raw data files (.wiff) were converted to .abf format and processed via MS-DIAL (version 4.60) for peak picking, alignment, and annotation against the LipidBlast database. High-confidence identification was ensured by setting mass tolerances at 0.01 Da (MS1) and 0.05 Da (MS2) with an 80% score threshold. Background noise was filtered out by comparing sample peaks against the blank solvent injections. Furthermore, to ensure data stability and quantitative reproducibility, lipid features exhibiting a relative standard deviation (RSD) greater than 30% across the pooled quality control (QC) samples were systematically removed. Putative lipids underwent manual curation in PeakView (version 1.0) to validate mass accuracy (<5 ppm), isotopic distribution (<10%), and retention time logic, thereby removing unstable or artifactual features.

For lipid quantification, a semi-quantitative approach was adopted. Given the highly diverse nature of untargeted lipidomics, constructing individual absolute calibration curves for all 311 identified lipid species is technically unfeasible. Therefore, relative quantification was executed in MultiQuant (version 3.0.3) by normalizing peak areas of endogenous lipids against their respective internal standards, without the application of absolute calibration curves or response factors. The 13 isotopically labeled internal standards were selected to represent the major lipid subclasses. For lipid subclasses lacking a direct corresponding internal standard, semi-quantification was performed using the internal standard with the most similar chemical structure and retention behavior (a detailed mapping of lipid subclasses to internal standards is provided in Table S9). Consequently, all quantitative data reported herein represent semi-quantitative relative abundances rather than absolute concentrations.

### 3.6. Statistical Analysis

Experimental data were acquired from three biological replicates and are expressed as mean ± standard deviation (SD). Prior to multivariate modeling, missing values in the raw dataset, representing signals intermittently falling below the instrumental limit of detection, were imputed by replacing them with 1/5 of the minimum positive value of each corresponding variable. Subsequently, the data matrix underwent Log10 transformation and Pareto scaling to approximate a normal distribution and reduce the influence of extreme values.

Unsupervised pattern recognition, including principal component analysis (PCA) and hierarchical clustering analysis (HCA), was performed using the MetaboAnalyst 6.0 web platform (http://metaboanalyst.ca) to evaluate global lipidomic variance. To further validate group separation, a supervised partial least squares discriminant analysis (PLS-DA) was executed utilizing the Metware Cloud platform (http://cloud.metware.cn). The predictive performance (Q2) of the PLS-DA model was evaluated using a 10-fold cross-validation method, along with a permutation test (n = 200) to rigorously assess model robustness and guard against overfitting.

To strictly pinpoint significantly differential accumulated lipids, pairwise comparisons were conducted. An unpaired Student’s *t*-test was employed to assess the statistical significance of the differences between groups. Orthogonal partial least squares-discriminant analysis (OPLS-DA) was utilized via MetaboAnalyst 6.0 to calculate the variable importance in projection (VIP) values. The multidimensional screening criteria for extracting differential lipids were strictly set as VIP > 1.0, Fold Change (FC) > 2.0 or < 0.5, and FDR-adjusted *p* < 0.05 (using the Benjamini–Hochberg method to control the false positive rate). Finally, a Venn diagram was generated using the Metware Cloud platform to intersect the differential lipid sets, and boxplots were constructed to visualize their semi-quantitative abundance variations across different botanical origins.

### 3.7. Metabolic Pathway Analysis

Pathway enrichment and topological analyses of the differentially accumulated lipids were executed using the MetaboAnalyst 6.0 web portal. Given the structural complexity of intact lipid species, they were first manually mapped to their corresponding generic KEGG identifiers prior to analysis (e.g., mapping specific triacylglycerol species to the generic TAG KEGG ID: C00422). To prevent artificial inflation of pathway impact scores, if multiple lipid species mapped to the same generic KEGG identifier, they were treated as a single hit for topological calculations.

The statistical significance of pathway enrichment was determined via the hypergeometric test against the default background set, followed by the Benjamini–Hochberg false discovery rate (FDR) correction to strictly control for multiple testing. Pathway impact values were calculated based on relative betweenness centrality algorithms. Due to the limited genomic annotation currently available for *Fritillaria* species in public databases, the *Arabidopsis thaliana* pathway library within the Kyoto Encyclopedia of Genes and Genomes (KEGG) was employed as the default botanical reference metabolome. However, we acknowledge that using this reference may present methodological limitations, as certain *Fritillaria*-specific metabolic nodes could remain unannotated.

## 4. Conclusions

In this study, we provided the first comprehensive phytochemical characterization of the lipidomes of three dominant cultivated *Fritillaria* taxa. A highly conserved qualitative lipidome encompassing 311 shared lipid species was identified. Interestingly, *Fritillaria* bulbs exhibited a unique “precursor-rich” profile compared to related Liliaceae species, highlighting extensive lipid turnover potentially influenced by both alpine environmental adaptation and post-harvest processing. Furthermore, chemometric analysis revealed that the three sampled groups possessed highly distinct quantitative lipid phenotypes, driven by significant variations in glycerolipid and glycerophospholipid metabolism. We identified a panel of differentially accumulated lipids, specifically trace-level TAGs and FAHFAs, which exhibited pronounced stepwise variations among the groups and may hypothetically contribute to the overall anti-inflammatory efficacy of the bulbs. While further large-scale studies controlling for strict cultivation variables are required to fully disentangle the genetic and environmental contribution to these variations, this exploratory work establishes a robust phytochemical baseline for the *Fritillaria* genus. These findings offer novel perspectives for future research into the lipid-mediated environmental adaptation and metabolic diversity of alpine medicinal plants.

## Figures and Tables

**Table 1:** Lipid content (semi-quantitative relative concentration, μg/g) by category and subclass in three *Fritillaria* taxa.

Lipid Categories	Lipid Subclasses	Number of Identified Lipid Molecules	FU (*F. unibracteata*)		FC (*F. cirrhosa*)		FW (*F. unibracteata* var. *wabuensis*)	
			Content (μg/g)	Relative Abundance (%)	Content (μg/g)	Relative Abundance (%)	Content (μg/g)	Relative Abundance (%)
Fatty acyls (FAs)	FFA	20	1343.28 ± 200.30	37.23%	1486.11 ± 96.15	37.77%	1770.59 ± 143.92	47.12%
	FAHFA	11	12.77 ± 0.26	0.35%	29.35 ± 6.95	0.74%	9.35 ± 0.20	0.25%
	NAE	15	231.70 ± 7.04	6.42%	247.86 ± 14.04	6.30%	225.56 ± 8.43	6.00%
	**Sum**	**46**	**1587.75 ± 196.54**	**44.00%**	**1763.32 ± 91.65**	**44.81%**	**2005.50 ± 135.70**	**53.37%**
Glycerolipids (GLs)	MAG	7	69.81 ± 2.92	1.94%	77.31 ± 4.69	1.96%	67.92 ± 2.72	1.81%
	DAG	19	138.69 ± 22.19	3.84%	104.72 ± 4.56	2.66%	182.85 ± 13.09	4.87%
	DGGA	3	3.88 ± 0.67	0.11%	6.17 ± 0.41	0.16%	4.95 ± 0.13	0.13%
	MGDG	8	10.24 ± 0.58	0.28%	15.93 ± 2.12	0.41%	10.78 ± 1.23	0.29%
	DGDG	9	68.24 ± 9.77	1.89%	109.34 ± 6.28	2.78%	91.07 ± 5.78	2.42%
	TAG	41	159.13 ± 17.91	4.41%	161.96 ± 4.78	4.12%	112.36 ± 18.94	2.99%
	TAG_EST	4	1.45 ± 0.30	0.04%	3.69 ± 0.99	0.09%	1.21 ± 0.14	0.03%
	**Sum**	**91**	**451.45 ± 44.69**	**12.51%**	**479.13 ± 18.68**	**12.18%**	**471.14 ± 30.53**	**12.54%**
Glycerophospholipids (GPs)	PA	8	408.78 ± 73.11	11.33%	375.98 ± 16.07	9.56%	232.71 ± 50.05	6.19%
	LPA	3	3.13 ± 0.09	0.09%	3.30 ± 0.50	0.08%	2.65 ± 0.25	0.07%
	PEtOH	19	117.49 ± 9.25	3.26%	63.48 ± 3.94	1.61%	58.97 ± 6.97	1.57%
	PMeOH	1	0.81 ± 0.27	0.02%	1.36 ± 0.19	0.03%	0.93 ± 0.14	0.02%
	PE	9	20.66 ± 2.49	0.57%	21.07 ± 1.22	0.54%	26.55 ± 4.87	0.71%
	LPE	4	17.78 ± 3.39	0.49%	27.80 ± 7.23	0.71%	33.06 ± 5.36	0.88%
	PC	8	22.70 ± 1.90	0.63%	20.77 ± 0.62	0.53%	19.29 ± 1.56	0.51%
	LPC	4	6.17 ± 0.30	0.17%	10.25 ± 1.22	0.26%	7.63 ± 0.37	0.20%
	PI	2	82.31 ± 7.89	2.28%	67.43 ± 7.40	1.71%	70.91 ± 6.37	1.89%
	PG	8	52.36 ± 10.58	1.45%	110.48 ± 41.59	2.81%	56.68 ± 10.69	1.51%
	HBMP	1	1.14 ± 0.40	0.03%	0.90 ± 0.54	0.02%	0.68 ± 0.06	0.02%
	**Sum**	**67**	**733.32 ± 67.60**	**20.32%**	**702.82 ± 53.02**	**17.86%**	**510.05 ± 74.01**	**13.57%**
Sphingolipids (SPs)	Cer	43	191.87 ± 12.47	5.32%	182.00 ± 8.27	4.62%	258.34 ± 11.74	6.87%
	HexCer	23	234.77 ± 19.51	6.50%	322.26 ± 17.10	8.19%	145.78 ± 21.96	3.88%
	**Sum**	**66**	**426.64 ± 28.53**	**11.82%**	**504.26 ± 22.74**	**12.81%**	**404.12 ± 26.41**	**10.75%**
Sterol Lipids (STs)	ASG	34	359.47 ± 28.75	9.96%	393.05 ± 70.41	9.99%	295.73 ± 6.68	7.87%
	SG	3	44.93 ± 6.20	1.25%	86.34 ± 13.89	2.20%	65.62 ± 3.43	1.75%
	SE	4	5.02 ± 0.79	0.14%	6.07 ± 0.74	0.15%	5.67 ± 0.68	0.15%
	**Sum**	**41**	**409.42 ± 33.10**	**11.35%**	**485.46 ± 77.84**	**12.34%**	**367.01 ± 8.76**	**9.77%**
**Total**	**26**	**311**	**3608.48 ± 64.09**	**100.00**	**3934.99 ± 148.25**	**100.00**	**3757.72 ± 170.81**	**100.00**

Values are presented as mean ± SD (n = 3); % represents the percentage of a certain subclass of lipids relative to the total lipid content.

## Data Availability

The original contributions presented in this study are included in the article/Supplementary Material. Further inquiries can be directed to the corresponding author.

## Supplementary Materials

The following supporting information can be downloaded at: https://www.mdpi.com/article/10.3390/plants15182782/s1.

## Abbreviations

The following abbreviations are used in this manuscript:

UPLC	Ultra-Performance Liquid Chromatography
Q-TOF	Quadrupole Time-of-Flight
MS	Mass spectrometry
FU	*Fritillaria unibracteata*
FC	*Fritillaria cirrhosa*
FW	*Fritillaria unibracteata* var. *wabuensis*
FA	Fatty acyl
GL	Glycerolipid
GP	Glycerophospholipid
SP	Sphingolipid
ST	Sterol lipid
FFA	Free fatty acid
FAHFA	Fatty acid ester of hydroxyl fatty acid
NAE	N-acyl ethanolamine
MAG	Monoacylglycerol
DAG	Diacylglycerol
TAG	Triacylglycerol
DGGA	Diacylglyceryl glucuronide
MGDG	Monogalactosyldiacylglycerol
DGDG	Digalactosyldiacylglycerol
TAG_EST	Triacylglycerol estolide
PA	Phosphatidic acid
LPA	Lysophosphatidic acid
PEtOH	Phosphatidylethanol
PMeOH	Phosphatidylmethanol
PE	Phosphatidylethanolamine
LPE	Lysophosphatidylethanolamine
PC	Phosphatidylcholine
LPC	Lysophosphatidylcholine
PI	Phosphatidylinositol
PG	Phosphatidylglycerol
HBMP	Hemibismonoacylglycerophosphate
Cer	Ceramide
HexCer	Hexosylceramide
ASG	Acylated steryl glycoside
SG	Steryl glycoside
SE	Sterol ester
SFA	Saturated fatty acid
MUFA	Mono-unsaturated fatty acid
PUFA	Poly-unsaturated fatty acid
PCA	Principal component analysis
QC	Quality control
PLS-DA	Partial least squares discriminant analysis
HCA	Hierarchical clustering analysis
OPLS-DA	Orthogonal Partial Least Squares Discriminant Analysis
ACN	Acetonitrile
IPA	Isopropanol
