# Supplementary material for: Untargeted Lipidomic Profiling Reveals Distinctive Lipid Features in Three Cultivated Fritillaria Species
Source: Plants (Basel). 2026 Sep 11;15(18):2782. doi: 10.3390/plants15182782 (PMC13610259; doi:10.3390/plants15182782)
Supplement: Supplementary file 1 [file plants-15-02782-s001.zip › Figures S1-S5.pdf]

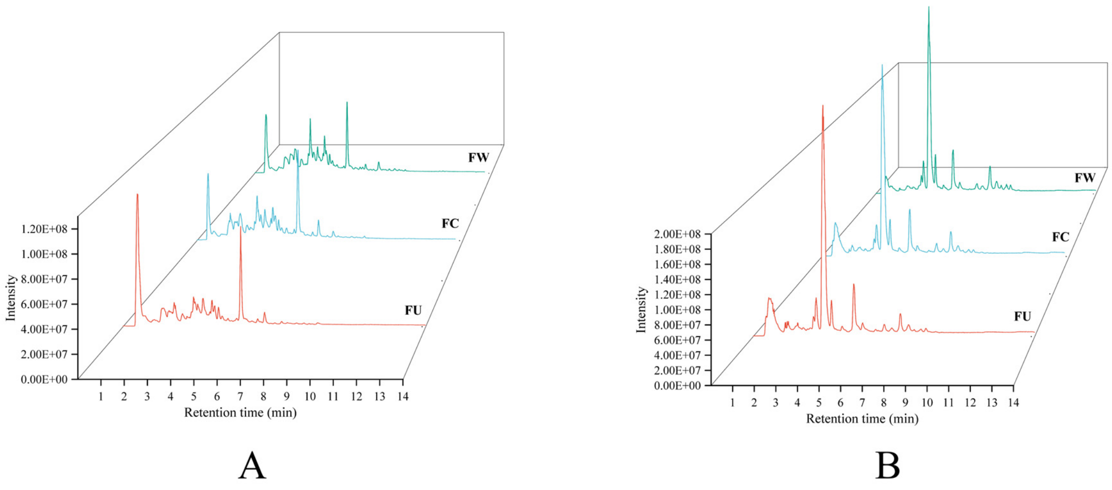


**Figure S1.** Total ion chromatograms of lipid molecular species of three *Fritillaria* species: (**A**) positive mode, (**B**) negative mode. FU: *F. unibracteata*, FC: *F. cirrhosa*, FW: *F. unibracteata* var. *wabuensis*.

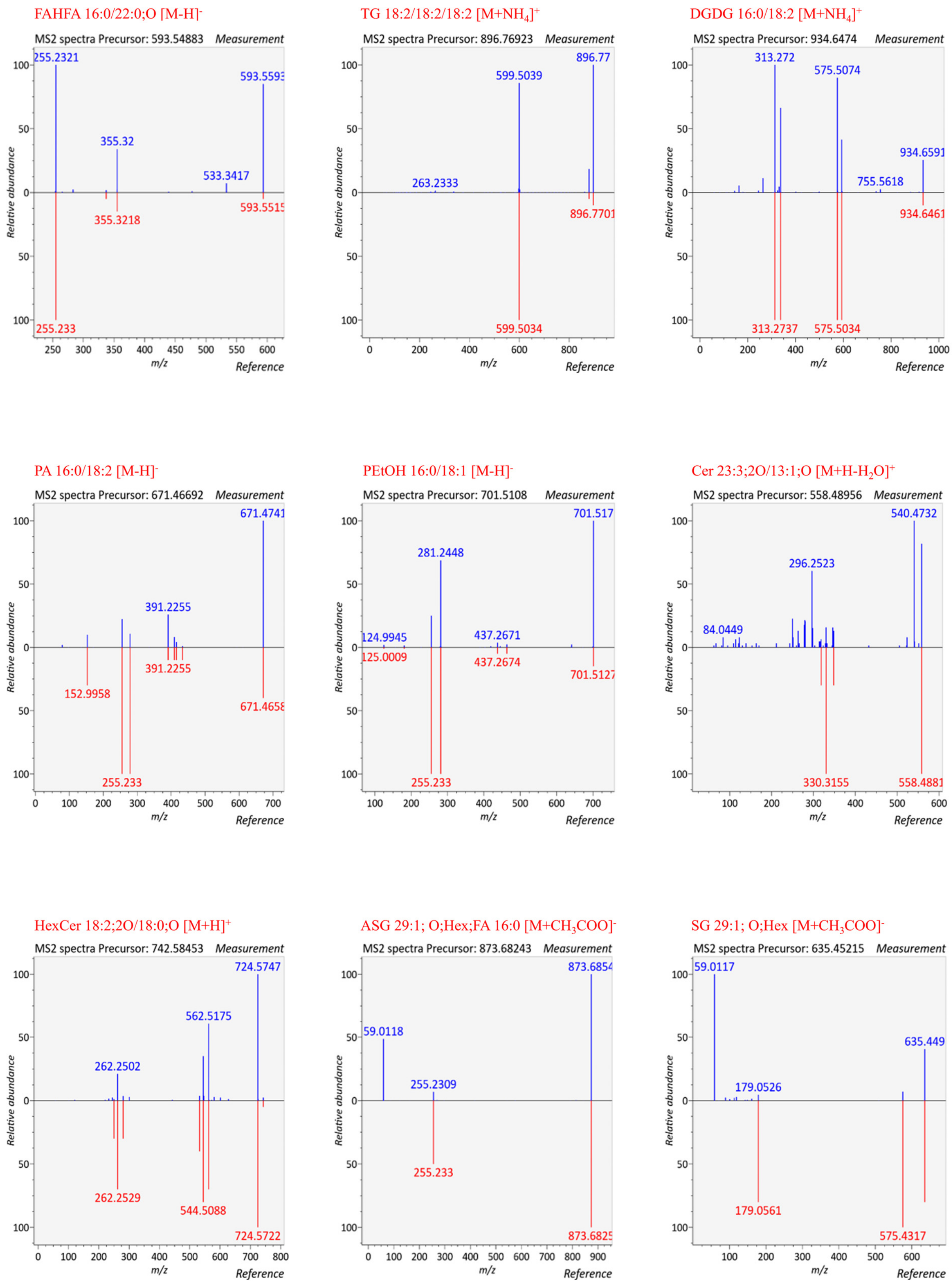


**Figure S2.** Some examples of lipid species identification via MS/MS spectra matching in MS-DIAL with integrated LipidBlast database.

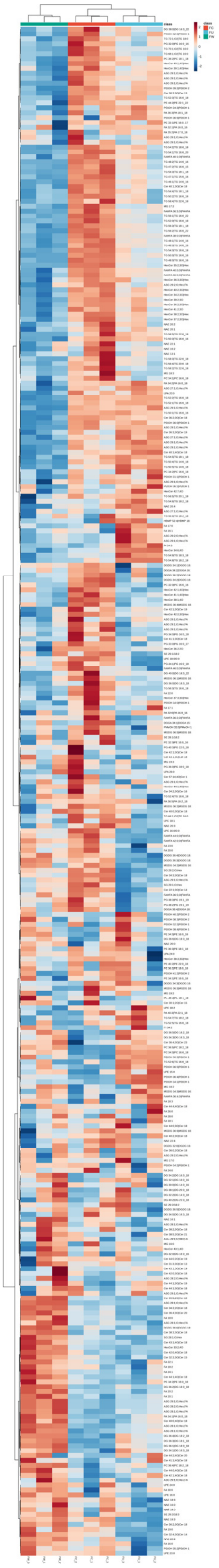

**Figure S3.** The heatmap for the hierarchical clustering analysis of the lipid profiles of three *Fritillaria* species. FU: *F. unibracteata*, FC: *F. cirrhosa*, FW: *F. unibracteata* var. *wabuensis*. The color of each cell corresponds to the concentration of the corresponding lipid molecule.

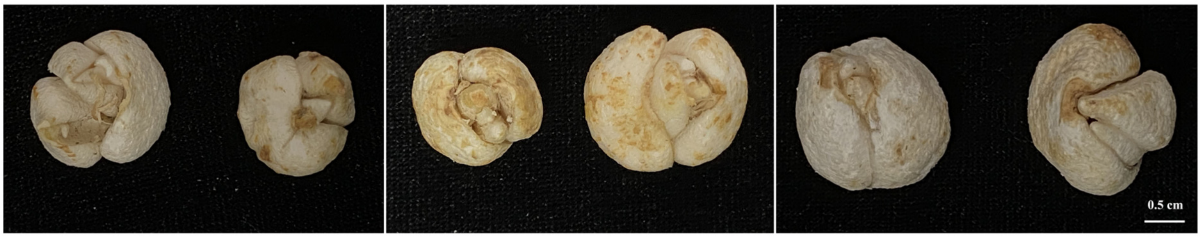


*F. unibracteata* (FU) *F. cirrhosa* (FC) *F. unibracteata* var. *wabuensis* (FW)

**Figure S4.** Morphological characteristics of bulb materials from three *Fritillaria* species.

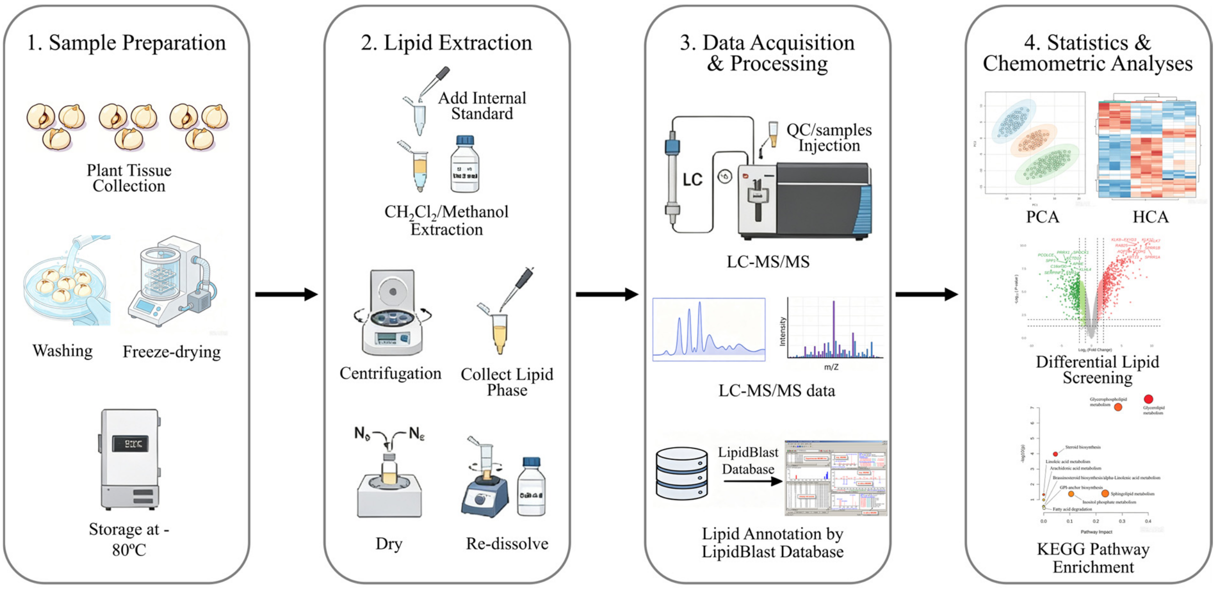


**Figure S5.** Schematic overview of the untargeted lipidomics workflow.
